# Unwelcome memento mori or best clinical practice? Community end of life anticipatory medication prescribing practice: A mixed methods observational study

**DOI:** 10.1177/02692163211043382

**Published:** 2021-09-08

**Authors:** Ben Bowers, Kristian Pollock, Stephen Barclay

**Affiliations:** 1 Primary Care Unit, Department of Public Health and Primary Care, University of Cambridge, Cambridge, UK; 2 Nottingham Centre for the Advancement of Research into Supportive, Palliative and End of Life Care, School of Health Sciences, University of Nottingham, Nottingham, UK

**Keywords:** Anticipatory prescribing, anticipatory medications, palliative medicine kit, terminal care, palliative care, mixed methods, end of life care, home palliative care, community nursing, general practice

## Abstract

**Background:**

Anticipatory medications are injectable drugs prescribed ahead of possible need for administration if distressing symptoms arise in the final days of life. Little is known about how they are prescribed in primary care.

**Aim:**

To investigate the frequency, timing and recorded circumstances of anticipatory medications prescribing for patients living at home and in residential care.

**Design:**

Retrospective mixed methods observational study using General Practitioner and community nursing clinical records.

**Setting/participants:**

329 deceased adult patients registered with Eleven General Practitioner practices and two associated community nursing services in two English counties (30 most recent deaths per practice). Patients died from any cause except trauma, sudden death or suicide, between 4 March 2017 and 25 September 2019.

**Results:**

Anticipatory medications were prescribed for 167/329 (50.8%) of the deceased patients, between 0 and 1212 days before death (median 17 days). The likelihood of prescribing was significantly higher for patients with a recorded preferred place of death (odds ratio [OR] 34; 95% CI 15–77; *p* < 0.001) and specialist palliative care involvement (OR 7; 95% CI 3–19; *p* < 0.001). For 66.5% of patients (111/167) anticipatory medications were recorded as being prescribed as part of a single end-of-life planning intervention.

**Conclusion:**

The variability in the timing of prescriptions highlights the challenges in diagnosing the end-of-life phase and the potential risks of prescribing far in advance of possible need. Patient and family views and experiences of anticipatory medication care, and their preferences for involvement in prescribing decision-making, warrant urgent investigation.

**What is already known on this topic?**

1. The prescribing of injectable anticipatory medications to provide symptom relief in the last days of life is recommended and widespread practice in a number of counties.
2. There is limited research concerning the frequency, timing and context of prescriptions.

**What this paper adds?**

1. Half (50.8%) of 329 patients whose deaths were potentially predictable deaths were prescribed anticipatory medications, the timing of prescriptions ranging from 0 to 1212 days before death (median 17 days).
2. Anticipatory medications were frequently prescribed as standardised drugs and doses, and often as part of a single end-of-life care planning intervention.
3. Patients’ and family carers’ involvement in prescribing decisions was unclear.

**Implications for practice, theory or policy**

1. Patient and family preferences for involvement in anticipatory medications prescribing decision-making and their experiences of care warrant urgent investigation.
2. The presence of anticipatory medications for long periods of time may compromise patient safety unless robust systems are in place to review their continued appropriateness and safe use.

## Introduction

Timely and effective symptom control in the last days of life is a key component in ensuring a comfortable death.^1–6^ In the UK, Australia, Canada, Norway and New Zealand, the individualised prescribing of injectable anticipatory medications, ahead of potentual need, for people approaching the end of life in the community is widely promoted to optimise symptom control in the last days of life at home and prevent unwanted hospital admissions.^7–13^ Anticipatory medications are kept in the home, where they are available to be administered by visiting nurses or doctors if the patient is unable to take oral medications and develops symptoms of pain, breathlessness, nausea and vomiting, agitation or respiratory secretions at the end of life.^7,14,15^

Although anticipatory prescribing is recommended practice in several countries^8,9,12,13^ there is inadequate evidence of its clinical effectiveness and limited research into the incidence and timing of prescriptions.^7,8^ Reported prescribing rates vary from up to 14% or 16% of predictable deaths in primary care populations in Australia and the UK^13,16^ to 63% of patients receiving specialist palliative care input.^17^ Most published evidence relates to UK practice.^7^ Patients with advanced cancer appear more likely to receive prescriptions than those with non-cancer terminal conditions.^11,18–20^ The timing of prescribing to death is reported as ranging from a few days,^11,19,20^ several weeks^15,17,19–21^ to several months before death.^22^

The decision to prescribe anticipatory medications is multifaceted and little studied to date. Community nurses report they initiate anticipatory prescribing conversations with patients and families when they perceive that death is imminent, following which they prompt General Practitioners (GPs) to prescribe medications.^15,23,24^ Nurses find some GPs to be resistant to prescribing anticipatory medications^18,23,25^ while other GPs act on their requests to prescribe.^13,23,24^ Some GPs prefer to independently judge when to prescribe anticipatory medications,^10,20,22^ or to discuss intended care with patients themselves and prescribe drugs whilst their condition is stable.^13,22^ Prescribing decisions involve assessing patient and family willingness to have end-of-life care discussions, safety risks associated with prescribing strong opioids and how soon medications may be needed.^15,20,22^

GP and community nurse records provide useful observational data for understanding practice. Retrospective examination of routinely collected clinical data enables investigation of recorded activities and interactions such as prescribing while avoiding selection and recruitment biases that are a major difficulty in prospective studies of terminally ill populations.^26,27^

Our study aims were to investigate the frequency, timing and recorded circumstances of injectable end-of-life anticipatory medications prescribing for patients living at home and in residential care.

## Methods

### Study design

We carried out a retrospective mixed methods observational study of deceased patients recorded care using GP and community nursing held clinical records.^28,29^ Quantitative and qualitative analysis were used to provide detailed and complementary insights into practice. Reflecting the social constructionist paradigm,^30^ clinical records are selective and stylised clinician accounts rather than presenting only objective facts.^31,32^

The Cambridge Positive Ageing and Cambridge Palliative and End of Life Care Patient and Public Involvement (PPI) Groups supported the study throughout. Both groups advised on the research priorities, the acceptability of accessing deceased patient records without consent and the interpretation of key findings.

### Ethical approvals

The South Cambridgeshire Research Ethics Committee granted ethics approval [Reference: 19/EE/0012]. The Health Research Authority’s Confidentiality Advisory Group [19/CAG/0014] approved the processing of confidential patient information without patient consent: data were anonymised at the earliest opportunity.

### Study population and setting

Participants were registered with eleven GP practices and two associated National Health Service Community Trusts providing community nursing services in two English counties. GP practices were purposively sampled from 21 practices expressing interest in participation, to obtain a maximum diversity sample in terms of patient list sizes (range 5500–43,000), geographical setting (two outer London practices, three urban, six rural town/villages) and practice population socioeconomic status (range third most deprived decile to the least deprived decile).

Each practice identified the 30 most recent expected deaths of patients aged 18+ years, who had been living at home or in residential care for at least 1 day in the last month of life and had died from any cause except trauma, sudden death or suicide. Patients living in nursing homes, with on-site trained nurses, were excluded as their care can differ considerably from those at home or in residential care. Patients who had previously indicated a wish not to be involved in research were excluded. Patients died between 4 March 2017 and 25 September 2019: one was excluded upon confirming their cause of death after data extraction, leaving a study population of 329 patients.

### Data sources and definitions

The electronic GP records and electronic and paper community nursing records of the deceased patients were retrieved and examined between May 2019 and March 2020 by BB, an experienced community and palliative care nurse. Patient demographic and clinical characteristics, documented end-of-life planning discussions and decisions, summary of events in the 7 days preceding anticipatory medication prescribing, recorded prescribing contexts and decision-making and medication details were entered into a custom-built secure database (Supplemental Document 1). Relevant free text record entries were summarised. Cause and date of death were confirmed from GP practice held death certificate books or England’s General Register Office.

Anticipatory medications were defined as one or more injectable medications prescribed ahead of need to be administered for symptom control in the last days of life.^7,14^

### Data analysis

Data analysis combined quantitative and qualitative analyses in a mixed methods approach.^28,29^ Categorical data are reported as frequencies and percentages and continuous data as median (interquartile range: IQR). The sample size of 330 patients was calculated a priori with a statistician to enable statistical analysis including Chi-square and Fisher’s exact test and multivariable logistic regression models.^29^ Data analysis was performed using SPSS version 26: *p* < 0.05 is considered statistically significant.

BB undertook qualitative analysis using inductive constant comparison incident-to-incident coding^28^ of extracted data from the clinical records of all patients prescribed anticipatory medications focussing on end-of-life discussions, prescribing contexts and associated patient and family interactions, using NVivo version 12. BB is a clinical academic and community palliative care nurse with experience of conducting qualitative analysis. Thematic patterns and variances in records, typologies of care and decisions in attributing significance to findings were discussed and refined with KP, SB and both PPI groups. These iterative steps informed the interpretative analysis.^28,33^

## Results

Most deceased patients were either aged between 75 and 84 years (92/329, 28%) or 85 years and older (124/329, 37.7%). The majority of deaths were from non-cancer conditions (193/329, 58.7%). However, the most frequently occurring cause of death was solid tumours. See Table 1.

**Table 1.** Demographics and clinical characteristics of deceased patients.

Age range	*n*	(%)
18–64	50	(15.2)
65–74	63	(19.1)
75–84	92	(28.0)
85+	124	(37.7)
Gender male	169	(51.4)
Usual place of care		
Home	299	(90.9)
Care home	30	(9.1)
Ethnicity		
White	294	(89.4)
Other	11	(3.3)
Not recorded	24	(7.3)
Cause of death		
Cancer: solid tumour	130	(39.5)
Cancer: haematological malignancy	6	(1.8)
Chronic heart disease	41	(12.5)
Dementia	15	(4.6)
Pneumonia	48	(14.6)
Chronic obstructive pulmonary disease	26	(7.9)
Stroke	13	(4)
Liver disease	2	(0.6)
Acute heart disease	3	(0.9)
Frailty of old age	22	(6.7)
Other	23	(7)

In total, 167/329 (50.8%) patients were prescribed anticipatory medications. There was a wide range of prescribing rates across the eleven GP practices, with a median of 14/30 patients (46.7%) (IQR 11–17/30 patients) and range 7/30 (23.3%) to 28/30 (93.3%). There was a highly statistically significant association between the GP practice patients were registered with and whether they were prescribed anticipatory medications (*p* < 0.001). Patients who died from cancer were more likely to be prescribed medications (67.6%) than those who died from non-cancer conditions (38.9%) (*p* < 0.001). See Table 2.

**Table 2.** Univariate analysis of relationships of patient characteristics and anticipatory medication prescribing.

Patient characteristics	Prescribed anticipatory medications (167)/Total (329)		
Gender		*X*^2^ (DF = 1) = 0.503	*p* = 0.478
Male	89/169 (52.7%)		
Female	78/160 (48.8%)		
Age range		*X*^2^ (DF = 3) = 1.345	*p* = 0.718
18–64	24/50 (48%)		
65–74	33/63 (52.4%)		
75–84	43/92 (46.7%)		
85+	67/124 (54%)		
Ethnicity		*X*^2^ (DF = 2) = 0.304	*p* = 0.859
White	150/294 (51%)		
Other	6/11 (54.5%)		
Not Recorded	11/24 (45.8%)		
GP practice ID no.		*X*^2^ (DF = 10) = 36.059	*p* < 0.001
One	13/30 (43.3%)		
Two	14/30 (46.7%)		
Three	14/30 (46.7%)		
Four	28/30 (93.3%)		
Five	19/29 (65.5%)		
Six	16/30 (53.3%)		
Seven	16/30 (53.3%)		
Eight	14/30 (46.7%)		
Nine	13/30 (43.3%)		
Ten	7/30 (23.3%)		
Eleven	13/30 (43.3%)		
Number of practice chronic disease registers patient was on		*X*^2^ (DF = 4) = 12.789	*p* = 0.012
0–1	12/43 (27.9%)		
2–3	64/128 (50%)		
4–5	48/88 (54.5%)		
6–7	27/43 (62.8%)		
8–13	16/27 (59.3%)		
Usual place of residence		*X*^2^ (DF = 1) = 0.728	*p* = 0.393
Care home	13/30 (43.3%)		
Home	154/299 (51.5%)		
Cause of death		*X*^2^ (DF = 1) = 26.452	*p* < 0.001
Cancer	92/136 (67.6%)		
Non-cancer	75/193 (38.9%)		
DNACPR form completed		Fisher’s exact test	*p* < 0.001
Yes	163/227 (71.8%)		
No	4/102 (3.9%)		
On GP practice palliative care register		*X*^2^ (DF = 1) = 120.321	*p* < 0.001
Yes	135/168 (80.4%)		
No	32/161 (19.9%)		
Received specialist palliative care		*X*^2^ (DF = 1) = 86.166	*p* < 0.001
Yes	117/148 (79.1%)		
No	50/181 (27.6%)		
Preferred place of death		*X*^2^ (DF = 1) = 186.118	*p* < 0.001
Recorded	152/178 (85.4%)		
Not recorded	15/151 (9.9%)		

All statistically significant variables in the univariate analysis in Table 2 were entered into a multivariate regression analysis, which revealed that after adjustment for gender, age range, GP practice, number of chronic disease registers on, usual place of residence and cause of death, the likelihood of being prescribed anticipatory medications was significantly higher for patients with a recorded preferred place of death (OR 34; 95% CI 15–77; *p* < 0.001) and for patients who had received specialist palliative care (OR 7; 95% CI 3–19; *p* < 0.001) (Supplemental Document 2). Preferred place of death was included in the logistic regression model as the most theoretically sound marker of end-of-life planning; a completed Do Not attempt Cardiopulmonary Resuscitation (DNACPR) form and/or inclusion on the GP practice palliative care register was not always associated with a record of explicit end-of-life care planning.

## Recorded prescribing decision-making

Anticipatory medications were frequently recorded as being prescribed as part of a single end-of-life planning consultation: for 111/167 (66.5%) of patients prescribed medications, the prescription (or discussion concerning prescription) occurred during the same consultation when preferred place of death and/or cardiopulmonary resuscitation discussions were first recorded. There were three typologies of prescribing contexts. For 78/167 (46.7%) of patients, anticipatory medications were prescribed in the context of *rapid deterioration*; end of life was recorded as being imminent, with rapid deterioration of physical strength over a few days, escalating symptoms and reduced ability to eat or drink. Some patients then recovered and stabilised. The second prescribing context was *clinical uncertainty*; 17/167 (10.2%) of patients were prescribed anticipatory medications in case their condition did not improve, alongside the prescription of oral antibiotics for potentially reversible infections:

*6 days before death, GP visits the patient and records: ‘Deterioration, taken to bed, refusing drinks for the last 1-2 days. Comfortable, responding to carers*. . . *Unclear if has a urine tract infection (UTI) or this is a pre-terminal event. . . Tried phoning family but no answer. Plan: Completed DNACPR form with agreement of carers. Issued anticipatory medications and oral antibiotics. Treat for UTI and encourage fluids’. Computer codes: preferred place of care and death is home, patient is ‘aware of prognosis’. [Patient 91, GP Practice ID Four]*

In contrast, 72/167 (43.1%) of patients were prescribed anticipatory medications as part of *longer-term forward planning*. This was when patients had a relatively stable physical function, but the focus of care had shifted to end-of-life support:

*292 days before death - GP visits the patient and records: ‘Recently seen in hospital by oncology and has been told has extensive metastatic disease. For palliative care. Increasing weight loss. Mood stable despite of diagnosis and poor prognosis. Plan: anticipatory medications and chart done. Add to end-of-life care register’. Computer codes ‘months prognosis’ and ‘aware of prognosis’. [Patient 287, GP Practice ID Nine]*

Recorded patient and family involvement in decisions to prescribe anticipatory medications were variable. No prescribing conversations were recorded for 69/167 of patients (41.3%). For a few patients (6/167, 3.6%), it was recorded that they did not want to discuss their prognosis or consider that they were dying at the time of prescribing anticipatory medications; clinicians still documented a preferred place of care and death in records. These patients lived alone, and prescribing decisions were framed as being in their best interests:

*27 days before death, GP visits patient and records: Three recent hospital admissions in the last month for congestive cardiac failure symptom management. ‘Can barely get out of bed and needing carer visits four times a day. . . Does not want to discuss their prognosis. . . States wants active treatment and not wanting to engage in end-of-life care planning. . . Lives alone . . . May need to go into palliative care mode swiftly. Plan: best set [prescribe] anticipatory medications now’. Computer codes: preferred place of care and death is home. [Patient 24, GP Practice ID One]*

Patient and family involvement in decision-making was recorded for 71/167 (42.5%) of patients, the records focussing on whether they agreed with clinician decisions to prescribe anticipatory medications: 10/71 patients (6%) were prescribed drugs prior to a visit or phone call to discuss prescribing. Most records concerning prescribing conversations were very brief, largely limited to reporting that families had been asked to collect the medications or patient/family agreement with prescribing decisions. More detailed anticipatory medication decision-making conversations were recorded when patients or families were concerned about possible symptoms (29/167, 17.4%). In a few cases (5/167, 3%), patient or families were recorded as not agreeing with a decision to prescribe anticipatory medications: three patients and one family were resistant to the idea of prescribing, and one patient was ‘aggrieved’ on discovering they had been prescribed medications without being asked. In these cases, clinicians recorded that it was in the patient’s best interests to have anticipatory medications available and documented persuading them to accept prescriptions:

*3 days before death, GP visits patient at home: ‘Discussed DNACPR and patient does not want resuscitation. States would like to pass away peacefully. . . Discussed anticipatory medications. [Patient states] does not want or need any medications. Explained these medications were only for if in distress. . . [Patient] remained adamant that does not need them. . . Discussed each group of medication and intended benefit in detail. . . I advised that they might not need them, and we will of course adhere to their wishes, but we do not want them to suffer. . . Agreed to [having] them’. [Patient 115, GP Practice ID Four]*

## Timing of prescribing

Anticipatory medications were prescribed between 0 and 1212 days before death. Patients who died from cancer were prescribed medications a median of 21.5 days before death (IQR 7–42 days, range 0–375 days); for those who died from non-cancer illnesses, medications were prescribed a median of 12 days before death (IQR 4–47 days, range 1–1212 days). Seven patients were prescribed anticipatory medications a year or more before death, of which six had a non-cancer diagnosis. The median prescribing timing was 17 days before death across the eleven GP practices, with range of median of 6–33 days between the practices. See Table 3.

**Table 3.** Timing of anticipatory medications prescribing in days before death.

GP practice ID no.	*n*	Minimum	Maximum	Median	IQR
One	13/30	0	375	17	9–78
Two	14/30	2	47	6.5	4.5–28.25
Three	14/30	2	374	17	8.5–36.75
Four	28/30	0	615	12.5	3–95
Five	19/29	1	695	33	6–60
Six	16/30	2	287	30	10–83.5
Seven	16/30	1	158	22.5	6.5–50.5
Eight	14/30	1	50	14	4.75–20.75
Nine	13/30	1	292	13	3.5–67
Ten	7/30	2	104	6	2–19
Eleven	13/30	3	1212	25	7.5–48.5

## Clinicians prescribing medications

For 71/167 (42.5%) of patients issued anticipatory medications, requests for prescriptions came from clinicians different to the prescriber: specialist palliative care team members (42/167, 25.1%), community nurses (20/167, 12%); GP practice-based paramedics (3/167, 1.8%); care home staff (2/167, 1.2%). GPs in all eleven GP practices (37/167, 22.2%) prescribed anticipatory medications following requests from specialist palliative care or community nurse colleagues without recorded contact with the patient or family.

The majority of anticipatory medications (127/167, 76%) were prescribed by GPs: other prescribers included hospital doctors (25/167, 15%), nurse prescribers (7/167, 4.2%), out of hours doctors (6/167, 3.6%) and specialist palliative care doctors (2/167, 1.2%).

## Symptom control prescribing

Most patients (154/167, 92.2%) were prescribed anticipatory medications for all five common end-of-life symptoms: pain, breathlessness, nausea and vomiting, agitation and respiratory tract secretions. Similar drugs and dose ranges were prescribed for all five symptoms following end-of-life electronic record template recommendations for 105/167 (62.9%) of patients. See Table 4.

**Table 4.** Anticipatory medications prescribed.

Drug group and name	*n*	Percentage (%)
Opioid	165	98.8
Morphine Sulfate^b^	114	68.3
Oxycodone^b^	26	15.6
Diamorphine^b^	25	15.0
Anxiolytic	166	99.4
Midazolam^a^	166	99.4
Anti-emetic	159	95.2
Haloperidol^c^	97	58.0
Cyclizine^c^	54	32.3
Levomepromazine^c^	5	3.0
Metoclopramide^c^	2	1.2
Ondansetron^c^	1	0.6
Antisecretory	163	97.6
Glycopyrronium^d^	152	91.0
Hyoscine Butylbromide^d^	9	5.4
Hyoscine hydrobromide^d^	2	1.2

The sample size was 167 for all drug groups. Recorded reason for prescription:

a Restlessness or agitation.

b Pain relief and shortness of breath.

c Nausea and vomiting.

d Respiratory tract secretions.

## Anticipatory syringe drivers (pumps)

For 49/167 (29.3%) of patients, a prescription for a continuous subcutaneous infusion of end-of-life care drugs was also issued ahead of need. These ‘anticipatory syringe drivers’ were usually for the same medications as anticipatory medication injections, often with larger dose ranges. The frequency and timing of anticipatory syringe driver prescriptions varied widely between GP practices, ranging from 1/16 patients (6.3%) to 10/14 patients (71.4%), with prescribing timing a median of 5.5 days before death across the eleven GP practices (range 2–27 days). See Table 5.

**Table 5.** Timing of anticipatory syringe driver prescribing in days before death.

GP practice ID no.	*n*	Minimum	Maximum	Median	IQR
One	3/13	6	94	27	–
Two	10/14	2	46	5.5	2.75–24.5
Three	7/14	1	18	2	2–9
Four	4/28	1	164	19.5	1.5–132
Five	4/19	1	36	2.5	1.25–27.75
Six	3/16	0	17	4	–
Seven	1/16	2	2	2	–
Eight	4/14	1	18	10.5	2–17.5
Nine	3/13	1	49	16	–
Ten	2/7	4	5	4.5	–
Eleven	8/13	2	536	15	3.5–102

## Discussion

Our study is the first to highlight the high frequency and standardised prescribing of anticipatory medication prescriptions for terminally ill patients in a primary care population. The range of timing of prescribing identified contrasts with the published evidence reporting that prescribing is limited to a few days to several weeks before death.^19,20^ Our findings correspond with GPs’ and nurses’ accounts of preferring to put anticipatory medications in place as early as is feasible to help manage any distressing symptoms in the final days of life.^15,21,22^

Prognostication is a very inexact science. It is difficult to predict the timing of death^34–37^ particularly for those with a highly unpredictable chronic frailty dying trajectory.^20,38,39^ Although anticipatory medications are typically prescribed closer to death for patients with non-cancer conditions,^19^ six of the seven patients in our study issued a prescription a year or more before death had non-cancer diagnoses. Some patients were prescribed anticipatory medications alongside antibiotics when there was clinical uncertainty about whether they were dying or had reversible infections. The prescribing of anticipatory medications can be perceived as an unwelcome reminder of death.^15,22^ The presence of the drugs in the home is also used by some visiting clinicians who are unfamiliar with the patient as a signal that care should focus on last days of life symptom control,^22^ even when this may not yet be the case.

Anticipatory syringe driver prescribing was common practice in several of the study GP practices. The recent Gosport War Memorial Hospital inquiry in the UK has highlighted the dangers for patient safety when prescribing anticipatory syringe drivers with large dose ranges to be started at the discretion of third parties whose clinical assessment skills are unknown to the prescriber.^7,40^ The inquiry found that at least 456 patients died where opioids had been prescribed often with the clinical instruction ‘please make comfortable’. These drugs were then administered in unjustified doses, commonly via syringe drivers.^40,41^ There is no previously published research on the practice of prescribing anticipatory syringe drivers: research is urgently needed to investigate the clinical appropriateness and safety of anticipatory syringe driver prescribing.^41^

Palliative care teams often initiate end-of-life care planning interventions including anticipatory medication prescription requests.^15,17,42^ Patients who had seen a specialist palliative care team were seven times more likely to be prescribed anticipatory medications. A referral to specialist palliative care, or the involvement of such a team, may again be perceived to be a signal to everyone involved that the patient is approaching end of life, which at times may not be the case.

End-of-life care planning is presented in current policy and clinical discourse as an evolving and individualised process that is started with patients whilst their condition is stable, with regular reviews as their situation and preferences change.^8,43–46^ In keeping with previous research, we found advance care planning decisions were frequently recorded as part of one main end-of-life care consultation or crisis intervention that comprises identifying preferred place of death, putting in place anticipatory medications and completing a DNACPR form.^22,47,48^ Primary care electronic end-of-life record templates, increasingly used to coordinate care across different services, aid communication and continuity of care.^26,37,49^ This technology also shapes practice and may inadvertently encourage the bureaucratisation of end-of-life care planning interventions by promoting a ‘one size fits all’ process.^22,32,50,51^ There is a tension between using templates to provide standardised guidance whilst promoting personalised care.

The preferences of clinicians and expectations of policymakers for ensuring that end-of-life advance care plans, including anticipatory medications, are in place, need to be balanced with patient and family readiness to have sensitive discussions and to make plans for future care.^8,44,47^ Our analysis found clinical records were largely silent about conversations with patients and family members concerning the implications and emotional impacts of anticipatory medication prescribing. Corresponding with previous research, there were occasions where professional led end-of-life planning, including the prescribing of anticipatory medications, took place without consultation with patients unwilling or unable to consider future care.^22,47,48^ Patient and family preferences for involvement in anticipatory medication prescribing decision-making, and their experiences of care, warrant urgent investigation.^7,22,52^

## Strengths and limitations

Caution is needed in interpreting what records can tell us about patient and family participation in prescribing decisions. Records only contain a small part of any clinical encounter, the emphasis frequently being on clinical decisions and prescribing matters.^32^ The lack of recorded information on patient and family understanding of anticipatory medications and their preferences is problematic in clinical practice as records are considered authoritative and influence subsequent care decisions.^31,32^ The research methods provide limited insights into patients’ and families’ perspectives and highlight that these are important aspects to explore. We have recently completed in-depth longitudinal interviews exploring patients’, families and their clinicians’ involvement in anticipatory medication decision-making (manuscript in preparation).

The generalisability of the results is enhanced by the identification of sequential deaths and purposive sampling of GP practices and community nursing services to obtain a maximum diversity sample of team cultures and practices.^28,29,53^ Rich descriptions of practice aid understanding and transferability of our results.^53^ Our methods enabled detailed qualitative and quantitative analysis of recorded events and their context, which would not have been possible through analysis of the available large primary care datasets.^26,49,54^ Anticipatory medication prescribing data, context and decision-making are not routinely recorded in a way which is systematically retrievable by using electronic algorithms. Consequently, details of care in the body of free text records are often overlooked in large database studies and valuable insights into practice are missed.^26,54^

We collected complete data from patient electronic clinical records: five patient community nursing paper prescription charts were missing. Prescribing events and contexts were confirmable from electronic records, and we present a full data set for all the variables analysed. As clinical records are not designed to collect research data, some patient characteristics were not routinely recorded. These include socioeconomic status, cohabitation status or perceived risks of opioids being misused or diverted, factors that may influence anticipatory medication prescribing.^20^ Data concerning the administration of anticipatory medications for these patients will be presented in a forthcoming paper.

## Conclusions

This mixed-methods clinical records study provides valuable insights into an important area of community end-of-life care practice. Standardised anticipatory medication prescribing patterns suggest undue reliance on electronic end-of-life care templates and a lack of individualised prescribing as advocated in international policy. Marked variability in the timing of prescriptions, at times many months before death, underscores the challenge of prognostication and highlights the risks involved in putting medication in place too far in advance of possible need. The presence of anticipatory medications for long periods of time, or when situations are uncertain, may therefore compromise patient safety unless robust systems are in place to review their continued appropriateness and safe use.

## Supplemental Material

sj-pdf-1-pmj-10.1177_02692163211043382 – Supplemental material for Unwelcome memento mori or best clinical practice? Community end of life anticipatory medication prescribing practice: A mixed methods observational study Click here for additional data file. Supplemental material, sj-pdf-1-pmj-10.1177_02692163211043382 for Unwelcome memento mori or best clinical practice? Community end of life anticipatory medication prescribing practice: A mixed methods observational study by Ben Bowers, Kristian Pollock and Stephen Barclay in Palliative Medicine

sj-pdf-2-pmj-10.1177_02692163211043382 – Supplemental material for Unwelcome memento mori or best clinical practice? Community end of life anticipatory medication prescribing practice: A mixed methods observational study Click here for additional data file. Supplemental material, sj-pdf-2-pmj-10.1177_02692163211043382 for Unwelcome memento mori or best clinical practice? Community end of life anticipatory medication prescribing practice: A mixed methods observational study by Ben Bowers, Kristian Pollock and Stephen Barclay in Palliative Medicine

sj-pdf-3-pmj-10.1177_02692163211043382 – Supplemental material for Unwelcome memento mori or best clinical practice? Community end of life anticipatory medication prescribing practice: A mixed methods observational study Click here for additional data file. Supplemental material, sj-pdf-3-pmj-10.1177_02692163211043382 for Unwelcome memento mori or best clinical practice? Community end of life anticipatory medication prescribing practice: A mixed methods observational study by Ben Bowers, Kristian Pollock and Stephen Barclay in Palliative Medicine

sj-xlsx-4-pmj-10.1177_02692163211043382 – Supplemental material for Unwelcome memento mori or best clinical practice? Community end of life anticipatory medication prescribing practice: A mixed methods observational study Click here for additional data file. Supplemental material, sj-xlsx-4-pmj-10.1177_02692163211043382 for Unwelcome memento mori or best clinical practice? Community end of life anticipatory medication prescribing practice: A mixed methods observational study by Ben Bowers, Kristian Pollock and Stephen Barclay in Palliative Medicine

sj-xlsx-5-pmj-10.1177_02692163211043382 – Supplemental material for Unwelcome memento mori or best clinical practice? Community end of life anticipatory medication prescribing practice: A mixed methods observational study Click here for additional data file. Supplemental material, sj-xlsx-5-pmj-10.1177_02692163211043382 for Unwelcome memento mori or best clinical practice? Community end of life anticipatory medication prescribing practice: A mixed methods observational study by Ben Bowers, Kristian Pollock and Stephen Barclay in Palliative Medicine
