# Supplementary material for: Unwelcome memento mori or best clinical practice? Community end of life anticipatory medication prescribing practice: A mixed methods observational study
Source: Palliat Med. 2021 Sep 8;36(1):95–104. doi: 10.1177/02692163211043382 (PMC8796157; doi:10.1177/02692163211043382)
Supplement: sj-pdf-2-pmj-10.1177_02692163211043382 – Supplemental material for Unwelcome memento mori or best clinical practice? Community end of life anticipatory medication prescribing practice: A mixed methods observational study [file sj-pdf-2-pmj-10.1177_02692163211043382.pdf]

Supplementary Document 2: Multivariable Logistic Regression

Supplementary Table 1. Prescribing of anticipatory medication: Multivariable Logistic Regression

							95% CI for Exp (B)	
Step 3a.	B	SE	Wald	df	Sig.	Exp (B)	Lower	Upper
**Gender** (female vs. male)	-0.091	0.382	0.057	1	0.811	0.913	0.431	1.931
**Age Range** (vs. 85+), years			2.426	3	0.489			
18-64	-1.005	0.657	2.342	1	0.126	0.366	0.101	1.326
65-74	-0.505	0.625	0.652	1	0.419	0.603	0.177	2.056
75-84	-0.213	0.511	0.174	1	0.677	0.808	0.297	2.199
**GP Practice ID No.** Reference = No. Eleven			12.366	10	0.261			
One	0.19	0.903	0.044	1	0.833	1.209	0.206	7.102
Two	0.554	0.973	0.324	1	0.569	1.74	0.259	11.714
Three	0.193	0.917	0.044	1	0.833	1.213	0.201	7.32
Four	2.632	1.099	5.738	1	0.017	13.9	1.614	119.75
Five	0.812	0.919	0.78	1	0.377	2.252	0.372	13.634
Six	-0.288	0.891	0.104	1	0.747	0.75	0.131	4.302
Seven	0.659	0.898	0.539	1	0.463	1.934	0.332	11.249
Eight	1.093	0.965	1.283	1	0.257	2.982	0.45	19.764
Nine	1.03	0.99	1.083	1	0.298	2.802	0.402	19.515

Ten	-0.345	0.996	0.12	1	0.729	0.708	0.101	4.986
**Number of chronic disease registers on** (vs. 8-13)			1.745	4	0.783			
0-1	-0.394	0.886	0.197	1	0.657	0.674	0.119	3.833
2-3	-0.225	0.715	0.099	1	0.753	0.799	0.197	3.244
4-5	-0.339	0.726	0.218	1	0.64	0.712	0.172	2.954
6-7	0.474	0.843	0.317	1	0.574	1.607	0.308	8.378
**Usual Place of Residence** (care home vs. home)	-0.023	0.699	0.001	1	0.974	0.977	0.248	3.842
**Cause of Death** (cancer vs. non-cancer)	0.27	0.517	0.273	1	0.601	1.31	0.475	3.61
**Preferred Place of Death** (recorded vs. not recorded)	3.533	0.416	72.266	1	0.000	34.238	15.16	77.322
**Received Specialist Palliative Care** (yes vs. no)	1.96	0.492	15.902	1	0.000	7.101	2.71	18.611
Constant	-3.028	1.107	7.49	1	0.006	0.048		

a. Variables entered: Gender, Age Range, GP Practice, Number Chronic Disease Register Groups, Usual Residence,

Cause of Death, Preferred Place of Death, Seen Specialist Palliative Care. Steps 1-3 are detailed in the Supplemental Document 3.
