# Supplementary material for: Unwelcome memento mori or best clinical practice? Community end of life anticipatory medication prescribing practice: A mixed methods observational study
Source: Palliat Med. 2021 Sep 8;36(1):95–104. doi: 10.1177/02692163211043382 (PMC8796157; doi:10.1177/02692163211043382)
Supplement: sj-pdf-3-pmj-10.1177_02692163211043382 – Supplemental material for Unwelcome memento mori or best clinical practice? Community end of life anticipatory medication prescribing practice: A mixed methods observational study [file sj-pdf-3-pmj-10.1177_02692163211043382.pdf]

Supplementary Document 3: Multivariable Logistic Regression Steps 1-3

Supplementary Table 2. Prescribing of anticipatory medication: Multivariable Logistic Regression Steps 1-3

	Step 1 / Model 1				Step 2 / Model 2				Step 3 / Model 3			
**Variable**	**B**	**SE**	**Sig.**	**OR**	**B**	**SE**	**Sig.**	**OR**	**B**	**SE**	**Sig.**	**OR**
Constant	.478	.697	0.493	1.613	-2.405	1.009	.017	.090	-3.028	1.107	0.006	0.048
**Gender**												
Female	-.070	.265	0.791	.932	-0.021	.363	0.953	.979	-0.091	0.382	0.811	0.913
(base = male)												
**Age Range, years**			0.120				0.775				0.489	
18-64	-0.821	.440	0.062	0.44	-0.57	0.611	0.351	0.566	-1.005	0.657	0.126	
65-74	-0.548	.405	0.176	0.578	-0.023	0.561	0.967	0.977	-0.505	0.625	0.419	
75-84	-0.775	.355	0.029	0.461	-0.057	0.487	0.908	0.945	-0.213	0.511	0.677	
(base = 85+)												
**GP Practice ID No.**			0.003				0.242				0.261	
One	-0.905	.603	0.133	0.405	-0.295	0.811	0.717	0.745	0.19	0.903	0.833	1.209
Two	-0.508	.606	0.402	0.602	0.381	0.837	0.649	1.464	0.554	0.973	0.569	1.74
Three	-0.375	.598	0.531	0.687	-0.016	0.82	0.984	0.984	0.193	0.917	0.833	1.213
Four	2.989	.882	0.001	19.872	2.747	1.099	0.012	15.597	2.632	1.099	0.017	13.9
Five	0.282	.640	0.659	1.326	0.631	0.846	0.456	1.88	0.812	0.919	0.377	2.252
Six	-0.193	.600	0.747	0.824	-0.472	0.782	0.546	0.624	-0.288	0.891	0.747	0.75
Seven	-0.255	.586	0.663	0.775	0.255	0.833	0.759	1.291	0.659	0.898	0.463	1.934
Eight	-0.365	.593	0.538	0.694	0.646	0.869	0.458	1.907	1.093	0.965	0.257	2.982
Nine	-0.518	.598	0.386	0.595	0.607	0.856	0.478	1.835	1.03	0.99	0.298	2.802
Ten	-1.211	.629	0.054	0.298	-0.428	0.875	0.625	0.652	-0.345	0.996	0.729	0.708
(base = No. Eleven)												
**Number of chronic disease registers on**			0.017				0.669				0.783	
0-1	-1.647	.650	0.011	0.65	-0.727	0.871	0.404	0.483	-0.394	0.886	0.657	0.674
2-3	-0.602	.529	0.255	0.529	-0.404	0.724	0.577	0.668	-0.225	0.715	0.753	0.799
4-5	-0.393	.534	0.462	0.534	-0.51	0.734	0.487	0.6	-0.339	0.726	0.64	0.712
6-7	0.293	.596	0.623	0.596	0.287	0.831	0.73	1.333	0.474	0.843	0.574	1.607
(base = 8-13)												
**Usual Place of Residence**												
Care Home	-0.471	0.515	0.36	0.624	-0.16	0.721	0.825	0.852	-0.023	0.699	0.974	0.977
(base = home)												
**Cause of Death**												
Cancer	1.861	0.311	.000	6.429	1.177	0.424	0.005	3.243	0.27	0.517	0.601	1.31
(base = non-cancer)												
**Preferred Place of Death**												
Recorded					3.799	0.398	.000	44.679	3.533	0.416	.000	34.238
(base = not recorded)												
**Received Specialist Palliative Care**												
Yes									1.96	0.492	.000	7.101
-2 Log likelihood	358.281				220.223				202.562			
	X2 = 97.734, df = 20, *p* <.001				X2 = 235.791, df = 21, p<.001				X2 = 253.453, df = 22, p<.001			
Nagelkerke R2	.343				.682				.716			
Hosmer and Lemeshow Test	*p* = 0.639				*p* =0.912				*p* =0.430			
Classification accuracy	72.9%				87.2%				88.8%			
